# Nanoribbon-Based Electronic Detection of a Glioma-Associated Circular miRNA

**DOI:** 10.3390/bios11070237

**Published:** 2021-07-13

**Authors:** Yuri D. Ivanov, Kristina A. Malsagova, Vladimir P. Popov, Tatyana O. Pleshakova, Andrey F. Kozlov, Rafael A. Galiullin, Ivan D. Shumov, Svetlana I. Kapustina, Fedor V. Tikhonenko, Vadim S. Ziborov, Alexander Yu. Dolgoborodov, Oleg F. Petrov, Olga A. Gadzhieva, Boris A. Bashiryan, Vadim N. Shimansky, Natalia V. Potoldykova, Dmitry V. Enikeev, Dmitry Yu. Usachev, Alexander I. Archakov

**Affiliations:** 1 Laboratory of Nanobiotechnology, Institute of Biomedical Chemistry, 119121 Moscow, Russia; kristina.malsagova86@gmail.com (K.A.M.); t.pleshakova1@gmail.com (T.O.P.); afkozlow@mail.ru (A.F.K.); rafael.anvarovich@gmail.com (R.A.G.); shum230988@mail.ru (I.D.S.); sveta.kapustina7.05@gmail.com (S.I.K.); ziborov.vs@yandex.ru (V.S.Z.); alexander.archakov@ibmc.msk.ru (A.I.A.); 2 Joint Institute for High Temperatures of Russian Academy of Sciences, 125412 Moscow, Russia; aldol@ihed.ras.ru (A.Y.D.); ofpetrov@ihed.ras.ru (O.F.P.); 3 Rzhanov Institute of Semiconductor Physics, Siberian Branch of Russian Academy of Sciences, 630090 Novosibirsk, Russia; popov@isp.nsc.ru (V.P.P.); ftikhonenko@gmail.com (F.V.T.); 4 Federal State Autonomous Institution “N. N. Burdenko National Medical Research Center of Neurosurgery” of the Ministry of Health of the Russian Federation, 125047 Moscow, Russia; ogadjieva@nsi.ru (O.A.G.); bbashiryan@nsi.ru (B.A.B.); Shimava@nsi.ru (V.N.S.); dousachev@nsi.ru (D.Y.U.); 5 Institute of Urology and Reproductive Health, Sechenov University, 119992 Moscow, Russia; potoldykovanv@gmail.com (N.V.P.); enikeev_dv@mail.ru (D.V.E.)

**Keywords:** nanoribbon, biosensor, silicon-on-insulator, glioma, early diagnosis, circular RNA, oligonucleotide

## Abstract

Nanoribbon chips, based on “silicon-on-insulator” structures (SOI-NR chips), have been fabricated. These SOI-NR chips, whose surface was sensitized with covalently immobilized oligonucleotide molecular probes (oDNA probes), have been employed for the nanoribbon biosensor-based detection of a circular ribonucleic acid (circRNA) molecular marker of glioma in humans. The nucleotide sequence of the oDNA probes was complimentary to the sequence of the target oDNA. The latter represents a synthetic analogue of a glioma marker—NFIX circular RNA. In this way, the detection of target oDNA molecules in a pure buffer has been performed. The lowest concentration of the target biomolecules, detectable in our experiments, was of the order of ~10^−17^ M. The SOI-NR sensor chips proposed herein have allowed us to reveal an elevated level of the NFIX circular RNA in the blood of a glioma patient.

## 1. Introduction

The majority of malignant brain tumors diagnosed in adults are gliomas. They include astrocytomas, glioblastomas (GBMs), oligodendrogliomas, and unspecified gliomas [1]. In particular, GBMs pertain to diffuse gliomas of astrocytic origin, being attributed to grade IV (according to the WHO classification [2]). That is, GBM is the most aggressive type of gliomas. It is also the most common type of malignant primary brain tumors, comprising 54% of all gliomas and 16% of all primary brain tumors [3]. To date, GBM is considered to be incurable. Among patients with diagnosed GBM, the mean survival rate makes up only fifteen months [4]. At present, a commonly used mode of GBM treatment consists of surgical resection of the tumor, followed by combined radiation therapy and temozolomide-based chemotherapy [5]. Furthermore, despite several authors considering small GBM tumors with a diameter of 8 mm to 14 mm as the early (or the initial) stage, the diagnosis was made after the progression of the tumor [6,7,8]. This indicates late diagnosis of GBM to be one of the causes of poor prognosis.

To date, neither standard diagnostic nor therapeutic strategies for the diagnosis of GBM at an early stage are developed. This is why an early revelation of GBM in humans represents an urgent problem of modern medical research, which still remains unresolved. In this respect, further development of methods based on liquid biopsy [9] and serological detection of GBM biomarkers [10] is a promising way towards early revelation of GBM. Early diagnosis of GBM will provide its timely treatment, thus improving the prognosis and the survival rate.

With account for cancerogenesis processes, a number of mechanisms are considered to be able to regulate the cancer development. Therefore, in recent decades, non-coding ribonucleic acids (ncRNAs) were acknowledged to play an important role in the development of oncological diseases [11]. As opposed to protein-encoding genes, ncRNAs can regulate gene expression either during the process of or after transcription, as well as in the course of translation. Circular ribonucleic acids (circRNAs), whose features are similar to those of ncRNAs, were recently considered as a new type of molecular markers of cancer [12].

CircRNAs pertain to the family of ncRNAs, representing single-stranded close molecules devoid of both the 5′-end cap and the 3′-end poly(A) tail formed by covalent binding. CircRNAs are represented in blood, being specific against the localization of diseases [13]. At the same time, they are considerably stable and can be considered as molecular markers for diagnosis and stage determination of various types of cancer (such as prostate, gastric, colorectal, and bladder cancer) [14,15], which is why circRNAs attract particular attention.

In addition, in recent years, several ncRNAs were found to be associated with cancer, including gliomas. With respect to studying ncRNAs, the expression pattern and characteristics of circRNA in gliomas attract growing interest [16,17]. By analyzing 46 samples, Song et al. [18] revealed 572 highly expressed circRNAs; of these, 476 were found to be expressed differentially between GBM and normal brain tissue, while the expression of 468 circRNAs in the normal tissue was significantly elevated in comparison with that in GBM tissue. Moreover, differentiated circRNA expression profiles were observed even in different anathomical points of normal brain tissue. These findings provided an important molecular and theoretical basis for tissue-specific expression patterns of circRNAs.

Circular RNA nuclear factor IX (circNFIX) was shown to participate in the development of many organs of the human body, including the brain [19,20]. Moreover, circNFIX can be associated with proliferation of glioma cells through the miR-34a-5p regulation [21]. That is, circNFIX was shown to be a molecular marker of glioma in humans.

The development of high-throughput RNA deep sequencing technology (RNA-seq) has allowed one to determine a widespread expression of circRNAs in human genes [22]. This was confirmed by quantitative polymerase chain reaction (qPCR) [23]. In this connection, it should be, however, emphasized that the implementation of RNA-seq requires considerable conceptual and computational improvements in order to increase the accuracy [24,25]. In general, polymerase chain reaction (PCR) has several disadvantages, and the extreme sensitivity to sample contamination is the most significant of them, since it often leads to obtaining false-positive results.

Thus, the correct timely diagnosis (that is, the early revelation) of GBM requires the development of novel techniques. With regard to serological analysis, this means the development of methods of circRNA biomarker detection with high concentration sensitivity of 10^−15^ M or better [26]. In this respect, molecular detectors, which allow one to detect single biological macromolecules, represent very attractive tools.

Molecular detectors allow one to count single target macromolecules, while not requiring their amplification [27]. In nanoribbon (NR) biosensors, the target molecules form complexes with the (nanoribbon surface)-immobilized probe molecules at the expense of biospecific affine probe–target interaction. These probe–target binding events cause a modulation of electric current, flowing through the NR, and the resulting electric signal is recorded. In this way, NR biosensors represent one of the types of molecular detectors [28,29,30,31], which provide label-free detection of target macromolecules in real time at subfemtomolar concentrations [32,33,34,35,36,37]. This allows one to consider NR biosensors as attractive tools for the early revelation of cancer in humans [38], when at least femtomolar (or better) detection sensitivity is required [26]. Nanoribbons (NRs) represent extended silicon nanostructures, which serve as the sensor elements of the NR biosensor. The length of the NRs is of the order of several microns, while their thickness is commonly within the nanometer range. This silicon NR is a component part of the “silicon-on-insulator” (SOI) nanostructure. The surface of this NR is exposed to the analyzed solution. Upon analysis of a sample, target molecules adsorb onto the NR surface (which serves as a virtual gate), modulating the electric current flowing through the NR [39,40]. In order to provide biospecific capturing of the target molecules, the NR surface is sensitized by covalent immobilization of molecular probes (probe molecules). For instance, DNA oligonucleotides, complimentary to the target nucleic acid molecules, can be employed as molecular probes. The formation of complexes with negatively charged macromolecules leads to an increase in the amount of an electric charge on the NR surface. This, in turn, leads to a decrease in the electric current, flowing through the NR, in the case of NRs with p-type conductance—or to an increase in the current if n-type NRs are employed. This current is registered in real time, and this is the principle of operation of the NR biosensor.

The NRs themselves have a high surface-to-volume ratio [41], and this is the key factor determining the extremely high sensitivity of the NR biosensor, which demonstrated single binding events of target biomolecules [30]. Moreover, one-dimensional morphology, in combination with a nanometer-scale size, promotes fast diffusion of the target molecules both towards and from the NR sensor surface. In this way, the target binding rate can be increased, thus providing faster sensor response and shorter recovery time [42]. This is why the development of smaller NRs represents a relevant task. In theory, the detection limit, attainable by using a NR biosensor, can be as low as several target molecules per sensor element [43].

In our present study, NR sensor chips, based on “silicon-on-insulator” (SOI) structures, have been developed. For the first time, we report the biosensor-based detection of circular RNA (circNFIX), which represent a molecular marker of glioma in humans, with SOI-NR sensor chips fabricated by CMOS-compatible technology. The target circNFIX RNA was isolated from real samples of plasma, obtained from glioma patients. The detection was performed in real time without introducing any additional labels for signal amplification. In this connection, it is to be emphasized that the recently reported methods of circular RNA detection are involving nucleic acid amplification [44,45], or require additional labels for signal amplification to be introduced [46]. Once again, herein, we consider the detection of a circular RNA—in contrast to microRNAs detection reported in our previous papers [29,35,36,37]. In the preliminary experiments, the detection of a synthetic deoxyribonucleic acid oligonucleotide (oDNA) analogue of circNFIX RNA in pure buffer at various concentrations has been performed in order to determine the lower detection limit attainable with our NR biosensor. In order to provide biospecific binding of the target molecules with the NR sensor surface, the latter has been sensitized with covalently immobilized DNA oligonucleotide molecular probes (oDNA probes), whose nucleotide sequence is known to be complimentary to the target molecules’ sequence. It has been demonstrated that the NR biosensor with oDNA-sensitized sensor elements, developed herein, can be successfully employed for the highly sensitive detection of their complementary oDNAs. The lower concentration limit of the target oDNA detection, attained in our experiments, amounted to 3.3 × 10^−17^ M. With the so-developed biosensor, an elevated circNFIX level in plasma of a glioma patient—in comparison with that in control plasma sample—has also been revealed.

## 2. Materials and Methods

### 2.1. Nanoribbon Biosensor

The NR biosensor represents a system which includes two main modules: the analytical module and the electronic measurement module [33,35].

**Figure 1:**
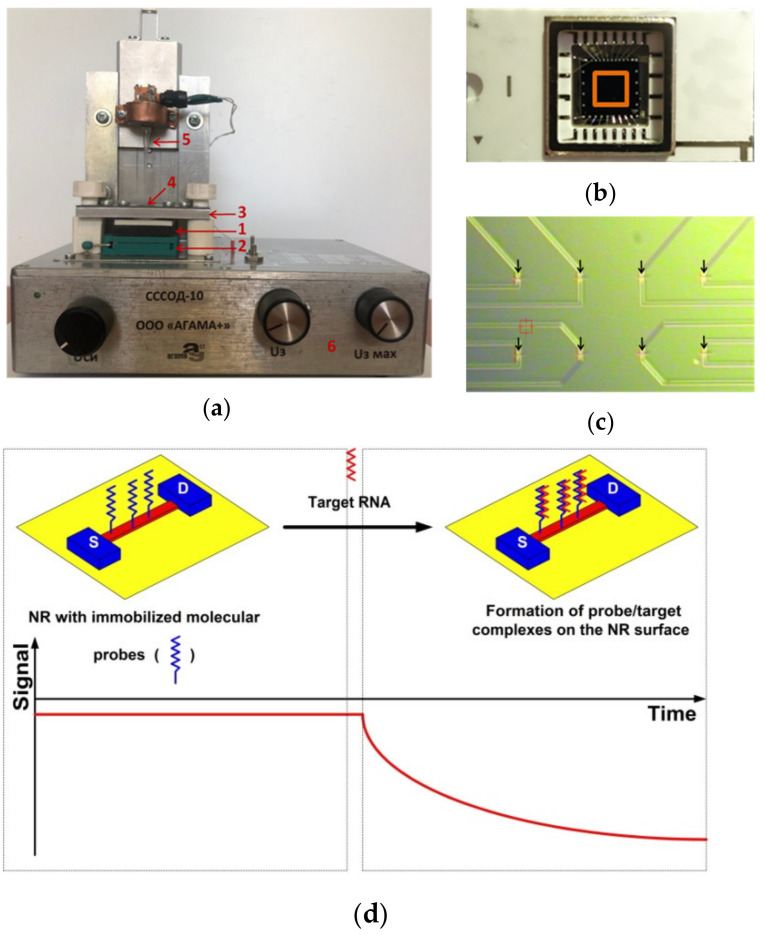
Photographic images of the entire analytical module of the NR biosensor (**a**), the SOI-NR sensor chip assembly (top view; red square indicates the sensitive area of the chip) (**b**), and the surface of the SOI-NR sensor chip with the array of NRs (arrows indicate the topography of the NRs) (**c**). Numbers indicate the main elements of the biosensor: 1—silicon-on-insulator nanoribbon sensor chip; 2—chip holder; 3—measuring cell holder; 4—measuring cell; 5—stirrer; 6—ten-channel data collection and storage system. Panel (**d**) displays a schematic of the principle of the real-time NR-based RNA detection. The conductance of the NR (and, accordingly, the biosensor signal) changes at the expense of the interaction between the NR-immobilized molecular probes and the target RNA molecules in the analyzed sample.

The analytical module comprises a 500-µL measuring cell, whose bottom is a SOI-NR sensor chip with an array of ten arranged-in-pairs NRs of n-type conductance (Figure 1a); the sensor chip is built-in into a standard microcircuit body (the sensor chip assembly; Figure 1b). A brief schematic of the NR-based RNA detection is shown in Figure 1d.

The electronic measurement module is intended for the simultaneous real-time registration and recording of the signal from the ten NRs, located on the sensor chip, and for the visualization of the recorded signal on the operator’s personal computer in the form of sensogram curves in the course of the experiment in real time. The digitization of the registered signal, its analysis, and the visualization of the results of the measurements was performed with the use of a specialized software (Agama + LLC, Moscow, Russia).

### 2.2. Fabrication of the SOI-NR Chips

**Figure 2:**
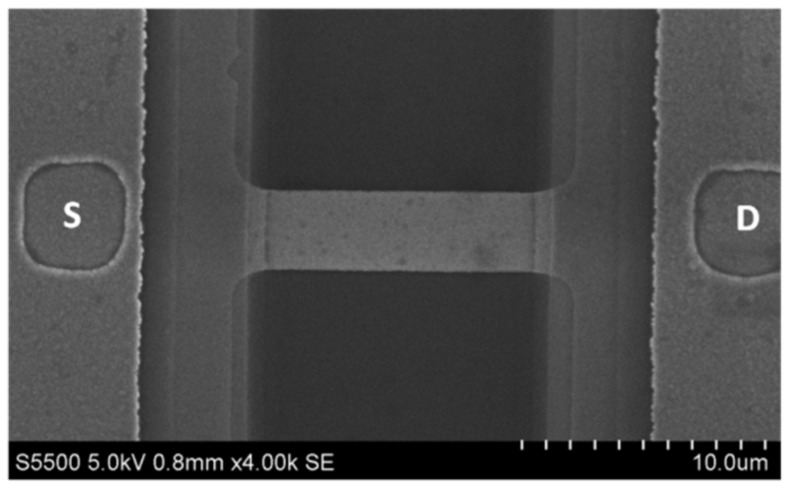
SEM image of an individual nanoribbon and ohmic contacts. The D and S letters indicate drain and source contacts, respectively.

The sensor chip assemblies were fabricated in Rzhanov Institute of Semiconductor Physics, Novosibirsk (https://www.isp.nsc.ru/en/, accessed on 10 July 2021), with the use of a technology analogous to Smart Cut. This technology includes transfer of silicon layers under the action of hydrogen onto the handle plate, but has several differences from the Smart Cut process. Namely, the buried oxide (BOX) is not subjected to hydrogen implantation (as in the Smart Cut process), and the interface between the BOX and the upper layer of silicon was a bonded interface. Such an approach to the SOI formation allows us to obtain Si/SiO_2_ systems with a low number of defects. This, accordingly, provides stability of the parameters of devices based on these systems. The technique of the SOI structure formation is described in detail elsewhere [47]. The chip contains structures, which comprise a sensor element (silicon nanoribbon) and drain and source ohmic contacts (Figure 2). The dimensions of the NRs on the chip crystal are as follows: width 3 µm, thickness 32 nm, length 10 µm (32 nm cut-off silicon layer thickness and 300 nm buried oxide layer thickness).

The formation of an array of 12 NRs on the SOI-NR chip determines their usability for multiplexed detection of several marker biomolecules during the analysis. In the experiments reported herein, SOI-NR structures with n-type conductance were employed.

### 2.3. Electrical Measurements

**Figure 3:**
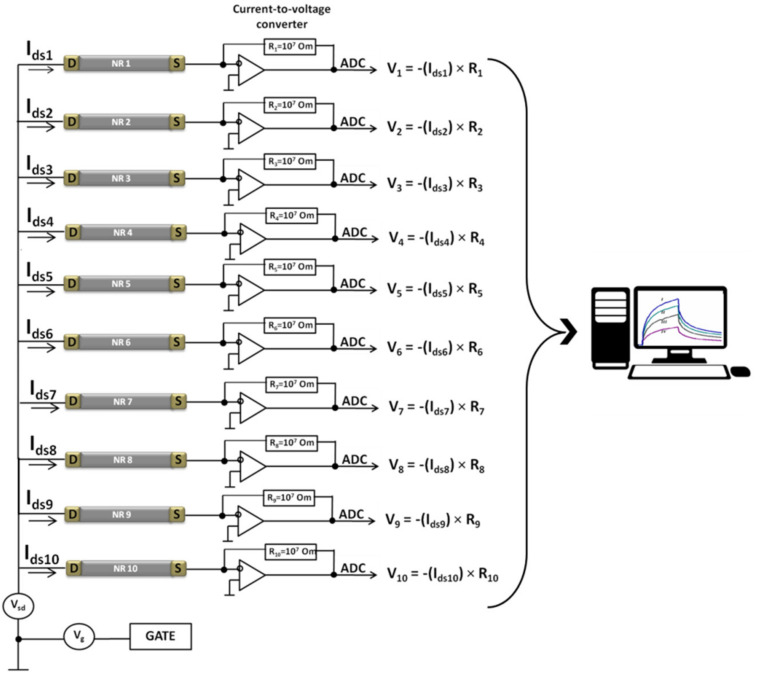
Electronic measurement circuit of the NR biosensor. Abbreviations: ADC, analog-to-digital converter; S, source; D, drain; *V_ds_*, drain–source voltage; *V_g_*, gate voltage; *I_ds_*, drain–source current; R, resistor; NR, nanoribbon.

Electrical measurements were performed with the use of a 10-channel data collection and storage system (Agama + JSC, Moscow, Russia). During the measurements, the substrate of SOI structures was used as a gate. The current flowing through the NRs was transduced into voltage with the use of current-to-voltage transducers, then digitized with an analog-to-digital converter, and displayed on the monitor of a personal computer (Figure 3).

Before the measurements, an operating gate voltage (*V_g_*), at which the drain-source current (*I_ds_*) is registered, is selected. The operating voltage is determined by measuring current-voltage characteristics, when the dependence of *I_ds_* on the supplied AC voltage (*V_g_)* is registered within the range from 0 to 60 V using a pure buffer. As a rule, a *V_g_* value within the subthreshold region is selected.

In the experiments with oDNA (a synthetic analogue of the circNFIX RNA), 150 µL of the analyzed oDNA solution in 1 mM potassium phosphate buffer (pH 7.4) were added into the measuring cell containing 300 µL of pure buffer. The operating parameters were as follows: gate voltage *V_g_* = 40 V, source–drain voltage *V_ds_* = 0.1 V. In the experiments on the detection of the circRNA, a 7 µL volume of the sample containing circRNA, isolated from the studied plasma, was added into the measuring cell containing 100 µL of 1 mM potassium phosphate buffer (pH 7.4), while using the following operating parameters: *V_g_* = 50 V, *V_ds_* = 0.1 V. The time dependencies of the drain-source current (*I_ds_(t)*) were recorded for each individual NR throughout the entire experiment. In our study, each NR was sensitized individually, as described below (Section 2.7 and Section 2.8). This approach has allowed us to form an array, containing working and control NRs, on the surface of a single NR sensor chip. The surface of the working NRs was sensitized by covalent immobilization of oDNA molecular probes complimentary to the target molecules. At the same time, the surface of the control NRs did not bear any probe molecules. The principle of the real-time RNA detection with the NR biosensor is schematically shown in Figure 1d. Capturing of target RNA molecules (which are charged negatively) onto the NR surface with immobilized molecular probes causes a decrease in the biosensor signal in the case when the NR represents an n-type nanotransistor.

The signal processing was performed in the following way. The registered changes in the level of the current signal *I_ds_* from each NR were normalized to 1 by dividing by the initial value of the current. Then, in order to account for the non-specific interactions, the values obtained in the blank experiment (i.e., with the use of a pure nucleic acid-free buffer instead of oDNA-containing solution) were subtracted from the data obtained during the analysis of the target oDNA solution. After that, a differential signal between the normalized signal from the working NR and that from control NR was calculated. The resulting time dependencies of the current signal *I_ds_*(*t*) were presented in the form of sensograms displaying differential signal, which was calculated by subtracting the signal from control SOI-NR from that of working SOI-NR.

### 2.4. Chemicals

Potassium phosphate monobasic (KH_2_PO_4_) and 3-aminopropyltriethoxysilane (APTES) were from Sigma-Aldrich (St.Louis, MA, USA). Isopropanol (C_3_H_8_O; 99.9% purity) was from Acros Organics (Geel, Belgium); hydrofluoric acid (HF) and ethanol (C_2_H_5_OH, 96% purity) were from Reakhim (Moscow, Russia). Moreover, 3,3′-dithiobis(sulfosuccinimidyl propionate) crosslinker (DTSSP) was from Pierce (Waltham, MA, USA). All solutions, used throughout the experiments, were prepared using deionized ultrapure water (of 18.2 MΩ/cm resistivity) obtained with a Simplicity UV system (Millipore, Molsheim, France).

### 2.5. DNA Oligonucleotides and Circular RNA

The sequence of oDNA probes, used for the sensitization of the NR sensors, was as follows: 5′-NH_2_-(T)_10_-TCCAGTTCTTTGATTGTGACTCCAATGTGATGTGGCTGGACGCACAGGCC. The oDNA probes were purchased from Evrogen (Moscow, Russia). The sequence of the NR-immobilized oDNA probes is complementary to the target oDNA sequence (GGCCTGTGCGTCCAGCCACATCACATTGGAGTCACAATCAAAGAACTGGA), which corresponds to that of NFIX circular RNA (hsa_circ_0005660). The surface of control NRs was free from immobilized oligonucleotides.

**Figure 4:**
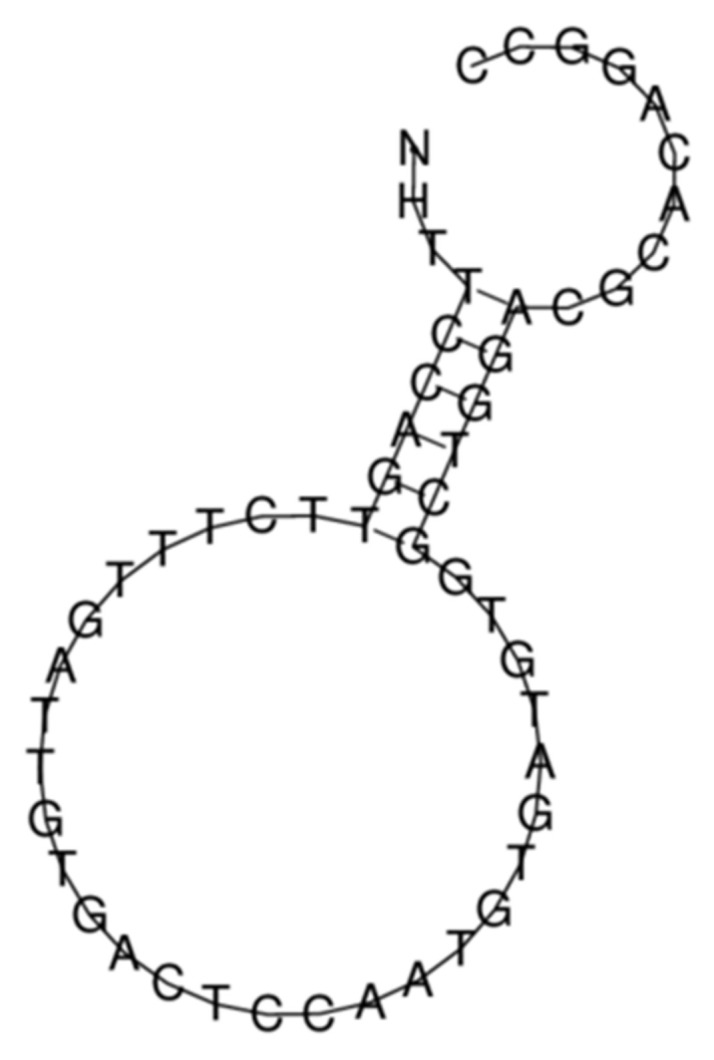
The chemical structure of NFIX circular RNA (hsa_circ_0005660) obtained using the RNAfold web server (http://rna.tbi.univie.ac.at/cgi-bin/RNAWebSuite/RNAfold.cgi; accessed on 30 June 2021).

Figure 4 displays chemical structure of hsa_circ_0005660 RNA obtained using the RNAfold web server (http://rna.tbi.univie.ac.at/cgi-bin/RNAWebSuite/RNAfold.cgi; accessed on 30 June 2021). From Figure 4, one can see that the target circular RNA has a centroid secondary structure.

The thermodynamic characteristics of this structure are as follows: the free energy of the thermodynamic ensemble is −10.43 kcal/mol; the frequency of the MFE structure in the ensemble is 7.06%; and the ensemble diversity is 14.76.

### 2.6. Plasma Samples

Plasma samples, used in our experiments, were obtained from patients with diagnosed glioma (Samples #001 and #002), examined in the Federal State Autonomous Institution “Scientific Research Institute of Neurosurgery named after Academician N.N. Burdenko” of the Ministry of Health of the Russian Federation (Moscow, Russia). As a control sample, plasma of a prostatic hyperplasia patient (Sample#14), examined in Institute of Urology and Reproductive Health (Sechenov University), was used. Table 1 summarizes the data on the plasma samples used in the experiments.

The plasma samples were taken according to the procedure described in our previous paper [35]. The samples were frozen and stored at −70 °C prior to the analysis.

Circular RNAs were extracted from the analyzed plasma samples with the use of the miRCURY™ RNA Isolation Kit—Biofluids (Exiqon A/S, Vedbaek, Denmark) in accordance with the manufacturer’s protocol.

### 2.7. Chemical Treatment of the SOI-NR Chip Surface

In order to clean the SOI-NR chip surface from organic contaminants, it was treated with aqueous isopropanol. In order to remove the native oxide layer from the sensor surface, a solution containing HF and ethanol was then applied, and after that, the SOI-NR chip was placed into an ozonator (UV Ozone Cleaner–ProCleaner™ Plus, Ossila Ltd., Sheffield, UK). In this way, hydroxyl groups were formed on the sensor surface. This allowed for its further silanization with APTES [32]. The silanized surface of the SOI-NR chip was finally washed with EtOH and dried.

### 2.8. Covalent Immobilization of the oDNA Probes

oDNA molecular probes, complimentary to the target biomolecules, were immobilized onto the surface of the SOI-NR sensors via DTSSP crosslinker as described in our previous papers [32,33,35,36]. Sensitization of individual SOI-NR sensors was achieved by using a Piezorray non-contact low-volume dispensing system (Perkin Elmer, Inc., USA), which allowed us to dispense as low as ~3 nL of solutions of oDNA probes onto the DTSSP-activated surface of individual SOI-NR sensors.

### 2.9. Preparation of Solutions of Target oDNA in Purified Buffer

The solutions of target oDNA with concentrations from 3.3 × 10^−18^ M to 3.3 × 10^−15^ M were prepared from the initial 100 mM stock solution in 50 mM potassium phosphate buffer (pH 7.4) by serial ten-fold dilution with working 1 mM potassium phosphate buffer (pH 7.4). On each dilution step, the solution was incubated in a shaker for 30 min at 10 °C and 600 rpm. The so-prepared solutions were then immediately used in the experiments. It should be noted that in the biosensor experiments, the final concentration of the target oDNA was three times lower, since 150 µL of the oDNA solution was added to 300 µL of pure buffer in the measuring cell. That is, the final concentrations of the target oDNA in the cell were from 1.1 × 10^−18^ M to 1.1 × 10^−15^ M.

## 3. Results

### 3.1. oDNA Detection in Buffer

The surface of the NR sensors was sensitized with oDNA molecular probes, specific against (i.e., complimentary to) the target oDNA. The latter represented a synthetic analogue of circNFIX RNA. For this purpose, the SOI-NR sensor chip was silanized in APTES vapours, and after the silanization, oDNA probes were covalently immobilized onto the surface of individual NRs by their reaction with DTSSP-activated terminal amine groups, exposed on this surface. After the sensitization, the experiments on the biosensor-based real-time detection of the target oDNA analogues of circNFIX RNA in pure buffer were carried out.

**Figure 5:**
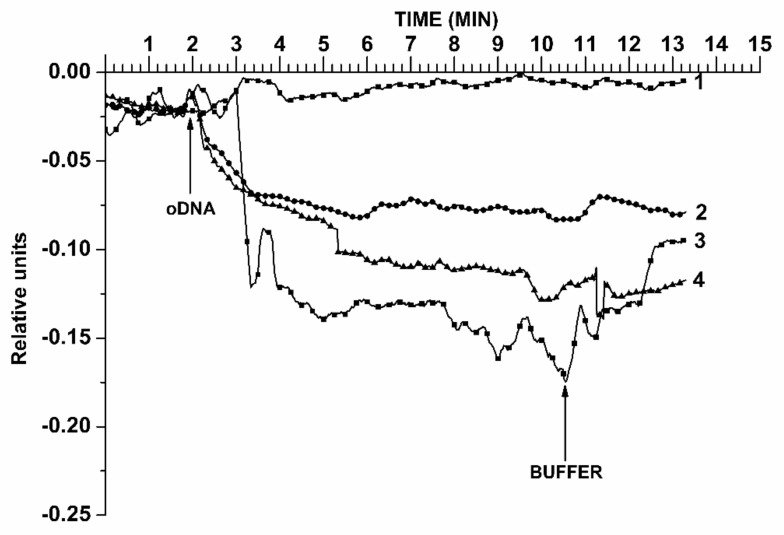
Typical sensograms obtained during the detection of oDNA analogue of circNFIX RNA in pure buffer solution, at various concentrations. Experimental conditions: target oDNA concentration 1.1 × 10^−18^ M (curve 1), 1.1 × 10^−17^ M (curve 2), 1.1 × 10^−16^ M (curve 3), or 1.1 × 10^−15^ M (curve 4); 1 mM potassium phosphate buffer (pH 7.4); *V_g_* = +40 V; *V_ds_* = 0.1 V. The total volume of the analyzed solution in the measuring cell was 450 µL. The time points of the addition of target oDNA solution and the wash of the sensor surface with pure potassium phosphate buffer are indicated by arrows.

The solutions of target oDNA with concentrations from 10^−18^ M to 10^−15^ M were added into the measuring cell (containing working buffer). In order to account for the non-specific binding, an oDNA-free NR (located on the same SOI-NR sensor chip), whose surface was free of any immobilized oDNA, was used as a control NR sensor. Figure 5 displays typical sensogram curves, obtained in the course of the analysis of the target oDNA in pure buffer at concentrations from 1.1 × 10^−18^ M to 1.1 × 10^−15^ M.

In Figure 5, curve 1 indicates the biosensor signal obtained upon analysis of 1.1 × 10^−18^ M oDNA solution. Moreover, one can clearly see that at such a concentration, the biosensor signal does not change. With a 10-times increase of the target oDNA concentration (to 1.1 × 10^−17^ M, curve 2), a change in the signal is observed. Namely, the signal decreases after the addition of 1.1 × 10^−17^ M oDNA solution. This is explained by the fact that the oDNA molecule is charged negatively owing to the content of phosphate groups. In the course of the biospecific capturing of the oDNA molecules from the analyzed solution onto the sensor surface, an increase in the electric charge density near the NR surface (which, in its turn, represents a virtual gate of the n-type field-effect transistor) occurs. Such an increase in the negative charge should lead to a decrease in the *I_ds_* current flowing through the NR. This is accompanied by the hybridization of the captured oDNA on the NR surface. After the analyzed solution was replaced with pure buffer (12th min), a slow dissociation of the probe/target complexes is observed. The slow dissociation indicates that the probe/target binding is considerably strong. The curves (1–4) presented in Figure 5 clearly indicate a decrease in the level of the biosensor response signal with decreasing the target oDNA concentration from 10^−15^ M to 10^−18^ M. In the control experiments, when oDNA-free buffer was added into the measuring cell instead of the oDNA solution, either no response from the NR sensors was detected, or the level of the biosensor response signal differed from the baseline level by <2%. These results indicate a biospecific interaction between the NR-immobilized oDNA molecular probes and the target oDNAs. Moreover, as one can see from the curves shown in Figure 5, in our experiments, the minimum detectable concentration of the target oDNA (representing a synthetic analogue of circNFIX RNA), amounted to 1.1 × 10^−17^ M.

### 3.2. Detection of circNFIX RNA, Isolated from Plasma

**Figure 6:**
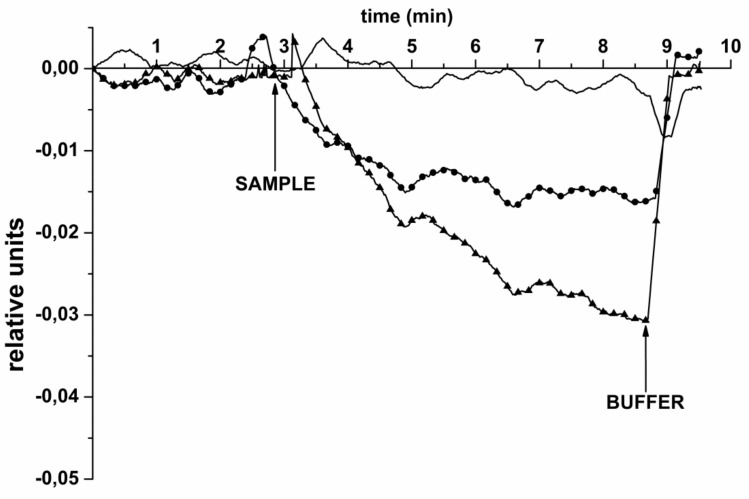
Typical sensograms obtained during the SOI-NR-based detection of circRNA, isolated from: the plasma of a healthy volunteer (control 1; no marker); the plasma of a prostatic hyperplasia patient (circles, ●); the plasma of a glioma patient (sample No. 001; triangles, ▲). Experimental conditions: 1 mM potassium phosphate buffer; Vg 50 V; Vds 0.1 V; total volume of the analyzed solution in the cell was 107 µL. The sensor surface was sensitized with oDNA probe #26. The time points of the addition of sample (circRNA solutions) into the cell, and washing with 1 mM fresh potassium phosphate buffer are indicated by arrows.

These experiments have been performed in order to determine whether circNFIX RNA—a molecular marker of glioma—isolated from plasma of glioma patients, can be revealed using a biosensor with oDNA-sensitized NRs. For this purpose, the same oDNA-sensitized NR sensor chip, as that described in the previous section, was employed. In control experiments, two types of samples were used: (1) circRNA isolated from the plasma of a healthy volunteer, and (2) circRNA isolated from the plasma of a prostatic hyperplasia patient. Figure 6 displays typical sensogram curves obtained in these experiments upon the SOI-NR-based detection of circNFIX RNA isolated from plasma samples.

The curves shown in Figure 6 clearly indicate a decrease in the biosensor signal after the addition of the sample containing circRNA, isolated from the plasma of a glioma patient (sample No. 001, circles)—as opposed to the case with the control sample, when the RNA was isolated from the plasma of a healthy volunteer (curve without marker). This corresponds to the expected increase in the level of the negative electric charge on the NR surface caused by the capturing of negatively charged circRNA. This is what causes the decrease in the electric current flowing through the field-effect nanotransistor. Namely, no change in the biosensor signal level was observed in the latter case. As regards the sample of a prostatic hyperplasia patient (circles), the level of the signal was significantly lower than that in the case of the sample of a glioma patient. The comparison of the sensograms shown in Figure 6, clearly indicates that our biosensor responds to the addition of the sample of a glioma patient much more intensively than to that of the sample of a hyperplasia patient. It should be emphasized that the detection of the target circNFIX RNA required less than ten minutes of time. This is in contrast to that required for the analysis of a sample by other methods, which amounts to several hours.

## 4. Discussion

Once again, our SOI-NR chips were fabricated on the basis of ”silicon-on-insulator” structures using a CMOS-compatible technology. The use of an array of oDNA-sensitized NRs on a single SOI-NR chip has allowed us to carry out rapid detection of target biomolecules in less than 15 min at concentrations ranging from 10^−17^ M to 10^−15^ M—that is, in lower femtomolar and subfemtomolar range. Rissin et al. emphasized the particular importance of this concentration range for the early revelation of cancer in humans [26]. This is especially actual in the case of glioma, since the latter is characterized by a low survival rate. Furthermore, the lowest concentration of the target biomolecules, detectable with the SOI-NR chips proposed, amounted to 1.1 × 10^−17^ M, while the time required for the analysis did not exceed 15 min. The lower limit of the circRNA detection of 1.1 × 10^−17^ M was determined on the basis of the following considerations. The signal obtained at 1.1 × 10^−18^ M oDNA concentration was virtually indistinguishable from the background signal. The signal at the oDNA concentration of 1.1 × 10^−17^ M exceeded the background signal by the value greater than 3σ. Accordingly, the 1.1 × 10^−17^ M oDNA concentration has been considered as the lowest concentration detectable with our biosensor. It is interesting to estimate the detectable quantity of miRNA in the volume of analyzed solution in the measuring cell of the biosensor upon the analysis. Given that the solution volume in the measuring cell amounted to 450 μL, the detectable number of the target molecules makes up

*N* = *C × V × N_A_* = 450 × 10^−6^ L × 1.1 × 10^−17^ M × 6.02 × 10^23^ molecules/mol = 2700 molecules,

where *C* is the lowest detectable circRNA concentration, *V* is the volume of the analyzed solution in the cell, and *N_A_* is the Avogadro number. The low detection limit is apparently determined by an increased local electrical conductance in the vicinity of the sensitive areas of the NRs owing to the cooperative effect of the near-surface water-(biological macromolecule) structure, as was noted elsewhere [48]. At the same time, let us note that at a 1.1 × 10^−18^ M concentration, the number of the target molecules amounts to 270, and our SOI-NR chip did not allow us to register such a low number of target molecules.

The high sensitivity of the nanoribbon-based detection is determined by the high surface-to-volume ratio of the NR sensor elements [41]. Moreover, the approach proposed herein was demonstrated to be suitable for rapid biospecific detection of target circular RNA in human plasma samples obtained from glioma patients. The conclusion on the biospecificity of the detection was based on the following criteria:

(1) Upon the registration of the biosensor signal in the experiments on the detection of oDNA in purified buffer solution at known target oDNA concentrations, the signal received from the working NR (whose surface is sensitized with immobilized molecular probes) should exceed that received from the control NR. The control NR is used in order to account for the non-specific adsorption, and the differential signal (i.e., the signal from the working NR minus the signal from the control NR) is used in the entire concentration range studied;
(2) In the experiments with plasma samples, the differential signal (the signal from the working NR minus the signal from the control NR), registered after the addition of the sample of a glioma patient, must exceed each of the differential signals obtained upon the analysis of (a) the sample of a healthy volunteer, and (b) the sample of a patient suffering from a different type of disease (in our case, from prostatic hyperplasia).

In our study, these two criteria have been satisfied, as shown in the Results section.

The applicability of NR sensors for the detection of cancer biomarkers in biological samples represents one more important feature, which makes these sensors attractive for the use in early diagnosis of cancer in humans [38]. The use of oDNA-sensitized SOI-NR chips has allowed us to reveal an elevated level of circNFIX RNA molecular marker of glioma in the blood of glioma patients. Thus, the biosensor with SOI-NR chips proposed herein represents a convenient tool for the rapid ultra-sensitive revelation of cancer-associated nucleic acid marker biomolecules in plasma samples.

## 5. Conclusions

Silicon-on-insulator structures-based nanoribbon sensor chips, sensitized with DNA oligonucleotides, have been developed. Using a synthetic DNA analogue of NFIX circular RNA marker of glioma, the concentration detection limit, attainable with the SOI-NR chips proposed, has been determined to be 3.3 × 10−17 M. The application of these chips for the real-time label-free detection of glioma-associated circular RNA, isolated from human plasma samples, has been demonstrated. The SOI-NR sensor chips developed have allowed us to reveal an elevated level of circNFIX RNA molecular marker in plasma samples of glioma patients—in comparison with that in the samples of healthy volunteer. The results obtained herein allow the SOI-NR biosensor to be considered as a promising tool for early revelation of cancer in humans.

## Figures and Tables

**Table 1:** Clinical and morphological characteristics of glioma patients and healthy volunteer.

Plasma Sample #	Age	Sex	Pathology	WHO Grade
001	67	M	Anaplastic oligodendroglioma	III
002	42	F	Anaplastic astrocytoma	III
14	73	M	Prostatic hyperplasia	-

## Data Availability

Correspondence and requests for materials should be addressed to Y.D.I.
